# A Walk on the Wild Side: The Impact of Music on Risk-Taking Likelihood

**DOI:** 10.3389/fpsyg.2017.00759

**Published:** 2017-05-10

**Authors:** Rickard Enström, Rodney Schmaltz

**Affiliations:** ^1^Department of Decision Sciences, MacEwan University, EdmontonAB, Canada; ^2^Department of Psychology, MacEwan University, EdmontonAB, Canada

**Keywords:** risk, music, consumer behavior, decision making, judgment

## Abstract

From a marketing perspective, there has been substantial interest in on the role of risk-perception on consumer behavior. Specific ‘problem music’ like rap and heavy metal has long been associated with delinquent behavior, including violence, drug use, and promiscuous sex. Although individuals’ risk preferences have been investigated across a range of decision-making situations, there has been little empirical work demonstrating the direct role music may have on the likelihood of engaging in risky activities. In the exploratory study reported here, we assessed the impact of listening to different styles of music while assessing risk-taking likelihood through a psychometric scale. Risk-taking likelihood was measured across ethical, financial, health and safety, recreational and social domains. Through the means of a canonical correlation analysis, the multivariate relationship between different music styles and individual risk-taking likelihood across the different domains is discussed. Our results indicate that listening to different types of music does influence risk-taking likelihood, though not in areas of health and safety.

## Introduction

Consumers’ risk perceptions and the impact they have on their behavior in the market place has received substantial attention in the marketing literature. Risk perceptions represent individuals’ subjective beliefs of both the likelihood of outcomes and the nature of the outcomes themselves. At a higher level, these deliberations can shed light on why people search for more information before making a purchase, why they develop a loyalty toward a brand, and why some people are more likely than others to adopt new products and services, including counterfeit brands (Veloutsou and Bian, 2008). Furthermore, when considering product and service options, consumers may use a number of strategies in an attempt to reduce the perceived risk (Brunel and Pichon, 2004). For instance, customers tend to regard high-quality warranties as a mean of reducing financial risk (Shimp and Bearden, 1982; Padmanabhan and Rao, 1993). Consumers have also been found to use extensive search efforts as a way to reduce perceived risk (Srinivasan and Ratchford, 1991; Dowling and Staelin, 1994). From the marketer’s point of view, the consumers’ perceived risk can be altered by investing in the branding of product or service (Aaker, 1991; Keller, 1993; Heilman et al., 2000; Erdem and Swait, 2004; Erdem et al., 2006).

Recently, and in stark contrast to the predictions of normative economics, the existence and stability of human preferences for risky choices have been questioned. This discussion revolves around the apparent incongruity of risk preferences across decision-making domains, context, and content; and, as a follow up, what decision-making and information processing strategies are employed and how people translate the prospects into latent idiosyncratic metrics. It is fair to say that the question regarding the existence and possible formation of risk preferences has yet to be resolved (Kusev et al., 2009; Fox and Tannenbaum, 2011). While there is noteworthy experimental research pertaining to the study of risk, most has emphasized the financial decision-making domain (e.g., Duclos et al., 2013). There has been little work done exploring the role of immediate environmental factors, such as music exposure, on the perception of risk.

Music surrounds us. In addition to our own choice of music listening, we are exposed to a myriad of music pieces every day in public spaces and commercial environments. There are estimates that we spend as much as 15% of our waking hours actively listening to music, not including what we hear while at a restaurant, shopping, or in the background of television, film and commercials (Rentfrow, 2012). In fact, music is present in some form in 30 to 40 percent of our daily lives (Juslin et al., 2008). Due to the vast technological development, resulting in portability and accessibility to music wherever we go, music has more or less become an ever-present component of our daily lives (Frith, 1996).

There has been a long-standing concern about the influence of music on risk-taking. From Elvis shaking his hips and supposedly driving adolescents to engage in promiscuous sex, to public outcry regarding the influence of artists like Marilyn Manson, music has anecdotally had an association with risky behavior (North and Hargreaves, 2008; Miranda, 2013). Much of the concern around music and risk has been speculative or based on correlational data, with a primary focus on fans of ‘problem music’ like rap and heavy metal (Roberts et al., 1998; Lacourse et al., 2001; Vogel et al., 2012). While there has been some research demonstrating that exposure to certain styles of music, regardless of preference, influence behavior both positively (e.g., Greitemeyer, 2011) and negatively (e.g., Timmerman et al., 2008), there has been little work looking at the immediate impact of music-listening on risk-taking likelihood.

### Present Study

The aim of our study was to explore the impact of exposure to different styles of music on the assessment of risk-taking likelihood. Specifically, we tested the impact of listening to different styles of music on the perception of risk-taking likelihood across five dimensions of risk-taking: financial, health and safety, recreational, ethical, and social decisions.

Participants were exposed to one of five different styles of music, or a control condition with no music present. The styles of music were based on the five-factor model of musical preferences (Rentfrow et al., 2011). Labeled MUSIC, these dimensions are Mellow, Unpretentious, Sophisticated, Intense, and Contemporary. The Mellow dimension is comprised of smooth and relaxing musical styles, the Unpretentious factor represents music in the country and singer-songwriter tradition, and the Sophisticated dimension subsumes music perceived as complex, intelligent, and inspiring. The Intense dimension relates to loud, forceful, and energetic music, while the Contemporary dimension encompasses rhythmic and percussive music. All of the music pieces presented to participants in this study were selected from the dimension-specific exemplars provided by Rentfrow et al. (2011).

## Materials and Methods

### Participants

A sample of 321 undergraduate students volunteered as part of an optional credit component toward an introductory psychology course at a university in Western Canada. This sample is drawn from the Department of Psychology subject pool with participants having an average age of 20.60 years and a standard deviation of 3.99 years. In this study, there are 11 variables considered in the analysis. After excluding participants who did not complete all sections of the study and participants who were suspicious of the procedure, the remaining sample amounted to 297, of which 224 were female and 73 were male. All of the participants provided written consent prior to their participation, and the study was approved by the University’s Research Ethics Board.

### Procedure

To control for demand effects, participants were told that the purpose of the study was to examine the impact of multitasking on cognitive performance, and that they would be listening to music, watching videos, or be exposed to some other form of distraction while completing a series of tasks. Following this, all participants learned that they had been assigned to the music condition. To allow participants time to become accustomed to the music, they were asked to wear a pair of headphones and complete a series of questionnaires before their cognitive performance would be measured. The questionnaires were given under the guise of pre-test personality measures. Included in the questionnaire was the Domain-Specific Risk-Taking (DOSPERT) scale (Weber et al., 2002; Blais and Weber, 2006; Hanoch et al., 2006). The DOSPERT is a validated and widely used scale that measures risk-taking likelihood on a 7-point scale (1 = extremely unlikely; 7 = extremely likely) across five domains: financial (e.g., investing 5% of your annual income in a very speculative stock), health and safety (e.g., riding a motorcycle without a helmet), recreational (e.g., going white-water rafting at high water in the spring), ethical (e.g., revealing a friend’s secret to someone else) and social decisions (e.g., starting a new career in your mid-thirties).

Participants were then randomly assigned to one of the music conditions or to the control condition. In the music conditions, participants listened to music pieces from one of the five latent music factors established by Rentfrow et al. (2011). The merit of using the model proposed by Rentfrow et al. (2011) is that it allows us to avoid subjective self-reports of music preference and provides a genre-free and validated dimensional approach to music. In all conditions, participants were told to wear headphones and that music would begin to play shortly. In the control condition, the music did not play. The experimenter remained blind to condition during data collection and only interacted with participants unblinded during the debriefing procedure. The music played for 1 min before participants began completing the questionnaires and continued while the participants completed the dependent measures. Upon completion of the measures, participants completed a funnel debriefing procedure. If a participant indicated that they were aware of the purpose of the study, his or her data was removed from the data set.

### Analysis

Participants’ responses to each of the five risk dimensions were first averaged so that the dependent variables were defined as participants’ average response to each of the five risk dimensions: Ethical, Financial, Health and Safety, Recreational, and Social. Input variables, in turn, were formed by coding each of the five music conditions, i.e., Unpretentious, Sophisticated, Mellow, Intense, and Contemporary, as dummy variables having the value of one if the condition occurred and zero if it did not occur. The no music condition constituted the comparison group. As there are several outcome variables, the multivariate relationship between the five music factors and the five risk-taking dimensions were analyzed through the means of a canonical correlation analysis (CCA). This approach allowed for a full disentanglement of the relationship between the two variable sets as well as coefficient estimates and signs.

## Results

To assess the multivariate relationship between the five music factors and the five risk-taking dimensions, a CCA was carried out. The analysis resulted in five canonical functions with squared canonical correlations of 0.822, 0.074, 0.014, 0.004, and 0.000, respectively. Jointly, the five canonical functions were statistically significant according to Wilks’ λ: λ = 0.162, *F* = 27.017, *p* < 0.000. Thus, the full canonical model across the five functions explained 83.8% of the shared variance between the two variable sets.

Looking at the dimensionality of the multivariate relationship between risk domains and music domains, the analysis revealed that in addition to the full model, the canonical functions 2 to 5 were statistically significant, *F* = 1.74, *p* = 0.035. However, canonical functions 3 to 5 and 4 to 5 were not statistically significant, *F* = 0.590, *p* = 0.806, and *F* = 0.333, *p* = 0.856, respectively. In addition, canonical function 5 did not explain a significant amount of the shared variance between the two variable sets, *F* = 0.083, *p* = 0.774. Given the hierarchical analysis, only the first two canonical functions were deemed practicable, thereby explaining 82.2 and 7.4% of the shared variance between the two variable sets, respectively.

**Table 1** presented below exhibits the canonical function coefficients for the first two canonical functions. Looking at the coefficients for the first function, one can establish that all music dimensions were significant and equally important in terms of explaining the independent canonical variate. For the dependent variable set, the Financial and Social risk-taking variables were both significant and roughly on par with each other in terms of contributing to the dependent canonical variate. All five music factors had coefficients with positive sign. Altogether, these findings indicate that, in comparison to the control group with no music, the exposure to all five music factors resulted in increased risk-taking with equal effect in the two significant risk-taking domains: Financial and Social.

**Table 1:** First and second canonical solution for music and risk domains.

	Function 1			Function 2		
Variable	Coefficient	*t*	*p*	Coefficient	*t*	*p*
Ethical risk	–0.029	–0.72	0.470	0.509	1.68	0.094
Financial risk	0.140	4.46	0.000	–0.273	–1.14	0.255
Health/Safety risk	–0.006	–0.21	0.833	0.434	1.90	0.059
Recreational risk	0.030	1.46	0.144	0.356	2.29	0.023
Social risk	0.124	5.10	0.000	–0.687	–3.73	0.000
Unpretentious	1.134	16.21	0.000	–1.073	–2.02	0.045
Sophisticated	1.076	18.20	0.000	0.318	0.71	0.480
Mellow	1.132	16.54	0.000	–1.195	–2.29	0.022
Intense	1.079	15.59	0.000	1.926	3.66	0.000
Contemporary	1.045	15.27	0.000	0.024	0.05	0.964

After extracting the first canonical solution, the second canonical solution suggests that three music factors Unpretentious, Mellow, and Intense mattered when describing the independent canonical variate. Of the three, Intense had almost twice as large effect size as Mellow and Unpretentious. Interestingly, while Intense exhibited a positive impact on the independent canonical variate, both Mellow and Unpretentious were negatively related to the independent canonical variate. For the dependent variable set, both Recreational and Social risk-taking were significant but with opposite sign with Social having roughly twice as large effect as the Recreational domain. Given the signs of the significant variables in the two variable sets, it can be concluded that the Unpretentious and Mellow music factors are positively related to Social risk-taking but negatively related to Recreational risk-taking. Most strikingly, however, the Intense music factor is positively related to Recreational risk-taking but negatively related to Social risk-taking. Consequently, the implications that follows are that when people are listening to Intense music such as heavy metal, they will be more inclined to engage in recreational activities often perceived as being risky, e.g., white water rafting or parachuting, in comparison to a situation when no music is being played. For the Social risk domain, however, the same music factor would instead decrease the likelihood of taking social risks; e.g., starting a new career or disagreeing with an authority figure on a major issue. Conversely, as people are hearing either Unpretentious or Mellow music, they will be more inclined to take social risks and less likely to take recreational risks.

## Discussion

While Intense music like punk, rap, and heavy metal has been correlated with risky behavior such as drug use and promiscuous sex, we found no relationship between listening to intense music and risk-taking likelihood. Listening to music does indeed impact risk-taking, but the picture is complex. Our results show that the exposure to intense music, or any type of music for that matter, does not impact either ethical or health and safety risk dilemmas. Listening to music, however, does impact the likelihood of engaging in social and recreational risk. To be specific, listening to Intense music, such as heavy metal or aggressive rap, increases risk-taking likelihood for recreational activities like bungee jumping off a tall bridge but reduces the tendency to take social risks such as starting a new career in your mid-thirties. Moreover, listening to either Unpretentious or Mellow music results in increased risk-taking in the social domain but in reduced risk taking in the recreational domain.

The media effects model may partially explain our results (Potter and Riddle, 2007). This model states that exposure to media, such as music, has an impact on our daily lives. In terms of music, the media effects model states that certain types of music act as a prime which can impact cognition and behavior in a manner that is congruent with the style or message of the music (Rentfrow, 2012). Our results are somewhat surprising in that listening to different styles of music did not uniformly impact risk. For example, listening to mellow music is positively related to social risk taking such as moving to a city far away from your extended family, while this same style of music is negatively related to recreational risks such as going camping in the wilderness.

The relationship between risk dimensions and music types is complex and multidimensional. Given our results, we argue that one possible explanation to the impact of music upon participants’ stated risk-taking intentions is that it functions as a soundtrack to people’s lives in a similar way as in motion pictures: mellow music in reflective scenes and intense music during exciting scenes. As it can be argued that some risk domains are more contemplative in nature and others are more charged, it follows that different music types could induce unique effects depending on the nature of the decision on hand. Regardless of the mechanism driving the effect, our research suggests that listening to music impacts consumer risk-taking likelihood.

Our future research will investigate the applicability of our results to the direct impact of music upon the evaluation of risk-related products and services. In particular, over and beyond the impact of music on information processing (Meyers-Levy and Zhu, 2010), certain styles of music should alter consumer purchase intentions of products or activities related to risk, such as bungee jumping or riding a motorcycle. Furthermore, longitudinal research exploring the impact of long-term exposure to different music styles would provide clarification on the lasting effects, if any, on risk perception.

## Ethics Statement

All subjects gave written informed consent in accordance with the Declaration of Helsinki. The protocol was approved by the MacEwan University Research Ethics Board.

## Author Contributions

RE is the lead author. Both authors RE and RS were involved in the data collection, analysis, and write-up of the manuscript for this paper.

## Conflict of Interest Statement

The authors declare that the research was conducted in the absence of any commercial or financial relationships that could be construed as a potential conflict of interest.

## References

[B1] Aaker D. (1991). *Managing Brand Equity: Capitalizing on the Value of a Brand Name*. New York, NY: The Free Press.

[B2] Blais A. R. Weber E. (2006). A domain-specific risk-taking (DOSPERT) scale for adult populations. *Judgm. Decis. Mak.* 1 33–47. 10.1002/bdm.414

[B3] Brunel O. Pichon P. E. (2004). Food-related risk-reduction strategies: purchasing and consumption processes. *J. Consum. Behav.* 3 360–374. 10.1002/cb.148

[B4] Dowling G. R. Staelin R. (1994). A model of perceived risk and intended risk-handling activity. *J. Consum. Res.* 21 119–134. 10.1086/209386

[B5] Duclos R. Wen Wan E. Jiang Y. (2013). Show me the honey! Effects of social exclusion on financial risk-taking. *J. Consum. Res.* 40 122–135. 10.1086/668900

[B6] Erdem T. Swait J. (2004). Brand credibility, brand consideration, and choice. *J. Consum. Res.* 31 191–198. 10.1086/383434

[B7] Erdem T. Swait J. Valenzuela A. (2006). Brands as signals: a cross-country validation study. *J. Market.* 70 34–49. 10.1509/jmkg.2006.70.1.34

[B8] Fox C. R. Tannenbaum D. (2011). The elusive search for stable risk preferences. *Front. Psychol.* 2:298 10.3389/fpsyg.2011.00298 PMC3216019 22110452

[B9] Frith S. (1996). “Music and identity,” in *Questions of Cultural Identity* eds Hall S. du Gay P. (Thousand Oaks, CA: Sage) 108–150.

[B10] Greitemeyer T. (2011). Exposure to music with prosocial lyrics reduces aggression: first evidence and test of the underlying mechanism. *J. Exp. Soc. Psychol.* 47 28–36. 10.1016/j.jesp.2010.08.005

[B11] Hanoch Y. Johnson J. G. Wilke A. (2006). Domain specificity in experimental measures and participant recruitment an application to risk-taking behavior. *Psychol. Sci.* 17 300–304. 10.1111/j.1467-9280.2006.01702.x 16623686

[B12] Heilman C. M. Bowman D. Wright G. P. (2000). The evolution of brand preferences and choice behaviors of consumers new to a market. *J. Market. Res.* 37 139–155. 10.1509/jmkr.37.2.139.18728

[B13] Juslin P. N. Liljeström S. Västfjäll D. Barradas G. Silva A. (2008). An experience sampling study of emotional reactions to music: listener, music, and situation. *Emotion* 8 668–683. 10.1037/a0013505 18837617

[B14] Keller K. L. (1993). Conceptualizing, measuring, and managing customer-based brand equity. *J. Market.* 57 1–22. 10.2307/1252054

[B15] Kusev P. van Schaik P. Ayton P. Dent J. Chater N. (2009). Exaggerated risk: prospect theory and probability weighting in risky choice. *J. Exp. Psychol. Learn. Mem. Cogn.* 35 1487–1505. 10.1037/a0017039 19857019

[B16] Lacourse E. Claes M. Villeneuve M. (2001). Heavy metal music and adolescent suicidal risk. *J. Youth Adolesc.* 30 321–332. 10.1023/A:101049212853

[B17] Meyers-Levy J. Zhu R. (2010). Gender differences in the meanings consumers infer from music and other aesthetic stimuli. *J. Consum. Psychol.* 20 495–507. 10.1016/j.jcps.2010.06.006

[B18] Miranda D. (2013). The role of music in adolescent development: much more than the same old song. *Int. J. Adolesc. Youth* 18 5–22. 10.1080/02673843.2011.65018

[B19] North A. C. Hargreaves D. J. (2008). *The Social and Applied Psychology of Music.* Cambridge: Cambridge University Press 10.1093/acprof:oso/9780198567424.003.0001

[B20] Padmanabhan V. Rao R. C. (1993). Warranty policy and extended service contracts: theory and an application to automobiles. *Market. Sci.* 12 230–247. 10.1287/mksc.12.3.230

[B21] Potter W. J. Riddle K. (2007). A content analysis of the media effects literature. *Journal. Mass Commun. Q.* 84 90–104. 10.1177/107769900708400107

[B22] Rentfrow P. J. (2012). The role of music in everyday life: current directions in the social psychology of music. *Soc. Personal. Psychol. Compass* 6 402–416. 10.1111/j.1751-9004.2012.00434.x

[B23] Rentfrow P. J. Goldberg L. R. Levitin D. J. (2011). The structure of musical preferences: a five-factor model. *J. Pers. Soc. Psychol.* 100 1139–1157. 10.1037/a0022406 21299309 PMC3138530

[B24] Roberts K. R. Dimsdale J. East P. Friedman L. (1998). Adolescent emotional response to music and its relationship to risk-taking behaviors. *J. Adolesc. Health* 23 49–54. 10.1016/s1054-139x(97)00267-x 9648022

[B25] Shimp T. A. Bearden W. O. (1982). Warranty and other extrinsic cue effects on consumers’ risk perceptions. *J. Consum. Res.* 9 38–46. 10.1086/208894

[B26] Srinivasan N. Ratchford B. T. (1991). An empirical test of a model of external search for automobiles. *J. Consum. Res.* 18 233–242. 10.1086/209255

[B27] Timmerman L. M. Allen M. Jorgensen J. Herrett-Skjellum J. Kramer M. R. Ryan D. J. (2008). A review and meta-analysis examining the relationship of music content with sex, race, priming, and attitudes. *Commun. Q.* 56 303–324. 10.1080/01463370802240932

[B28] Veloutsou C. Bian X. (2008). A cross-national examination of consumer perceived risk in the context of non-deceptive counterfeit brands. *J. Consum. Behav.* 7 3–20. 10.1002/cb.231

[B29] Vogel I. van de Looij-Jansen P. M. Mieloo C. L. Burdorf A. de Waart F. (2012). Risky music-listening behaviors and associated health-risk behaviors. *Pediatrics* 129 1097–1103. 10.1542/peds.2011-1948d 22614773

[B30] Weber E. U. Blais A. R. Betz N. E. (2002). A domain-specific risk-attitude scale: measuring risk perceptions and risk behaviors. *J. Behav. Decis. Mak.* 15 263–290. 10.1002/bdm.414

